# Cutaneous Larva Migrans among Devotees of the Nallur Temple in Jaffna, Sri Lanka

**DOI:** 10.1371/journal.pone.0030516

**Published:** 2012-01-25

**Authors:** Selvam Kannathasan, Arumugam Murugananthan, Nadarajah Rajeshkannan, Nilanthy Renuka de Silva

**Affiliations:** 1 Division of Parasitology, Department of Pathology, Faculty of Medicine, University of Jaffna, Jaffna, Sri Lanka; 2 Department of Community Medicine, Faculty of Medicine, University of Jaffna, Jaffna, Sri Lanka; 3 Department of Parasitology, Faculty of Medicine, University of Kelaniya, Ragama, Sri Lanka; Pennsylvania State University College of Medicine, United States of America

## Abstract

**Background:**

Many cases of Cutaneous Larva Migrans (CLM) have been observed among devotees, during and immediately after the annual festival at the Nallur Hindu temple in Jaffna.

**Objective:**

To ascertain the risk factors associated with infestation and devotees' knowledge and practices regarding the condition.

**Methodology/Principal Findings:**

A cross-sectional study using an interviewer-administered questionnaire and observation was conducted in August 2010. Out of 200 selected devotees 194(97%) responded. Soil and dog faecal samples were collected from the temple premises and examined for the presence of nematode larva and egg respectively. Among 194 male respondents, 58.2%(95% CI: 51.2%–65.0%) had lesions of CLM. One hundred and thirty (67%) respondents performed the ritual everyday; whereas 33% did so on special days. One hundred and twelve (57.7%) participants performed the ritual before 5.00am and remaining 42.3% performed after 5.00am. Among the participants, 77(36.7%) had the similar condition in previous years. One hundred and fifty seven (80.9%) were aware about this disease and 52(27%) devotees adopted some kind of precautionary measures. Bivariate analysis showed significant association between occurrence of CLM lesions and frequency of performing the ritual (p<0.001, OR-15.1; 95% CI:7.2-32.0), the timing of ritual performance (p = 0.022, OR-1.96; 95% CI:1.10–3.52), similar condition in previous year (p<0.001, OR-6.83; 95% CI: 3.39–13.76) and previous awareness of the condition (p = 0.005; OR-0.59;95% CI:0.43–0.82). Multivariate analysis showed that the frequency of ritual performance (OR-11.75; 95% CI 5.37–25.74) and similar conditions in previous years (OR-4.71; 95% CI: 2.14–10.39) were the independent risk factors. Two of the 20 soil samples were positive for the nematode larvae and three out of five dog faeces were positive for hookworm eggs.

**Conclusions/Significance:**

Deworming the stray dogs around the temple premises combined with the awareness programs among the public may be the effective and feasible precautionary measures to control similar epidemics in future.

## Introduction

Cutaneous larva migrans (CLM), also known as creeping eruption [1] of the skin, results from penetration of the human skin by parasitic larvae from domestic canine, bovine and feline hosts [1], [2]. Hookworms of dogs and cats, such as *Ancylostoma caninum* and *A. braziliense*, are the usual causative agents. Other nematode larvae, such as *Uncinaria stenocephala*, *Bunostomum phlebotomum* are also known as rare causative agents [3]. Temperature, humidity, porosity, texture, structure and consistency of the soil, wind, sunlight and the presence or absence of particular plants and animals are the physical, chemical and biological factors that influence the development and survival of the infective stages of hookworm larvae [4].

The typical clinical manifestation of this skin disease is an itchy, erythematous, linear or serpiginous, dermatitis tract [1], [3]. In most instances, the feet, buttocks and thighs [1] represent the main anatomical site affected by CLM, but any part of the body in contact with infested soil or sand can be involved [1], [2].

Although the prevalence of CLM are not widely documented in Sri Lanka, except two cases reported in 1980 among German travelers [5], in last few years, an unusually high number of cases of CLM have been observed in the Jaffna peninsula, in Northern Sri Lanka.

Since the above cases were specifically observed during and immediately after the Hindu festival celebrated at the Nallur temple in Jaffna, a preliminary study was carried out at this locality in 2001, which revealed a prevalence of 26.8% [6] among the 1014 devotees participated in this study.

**Figure 1:**
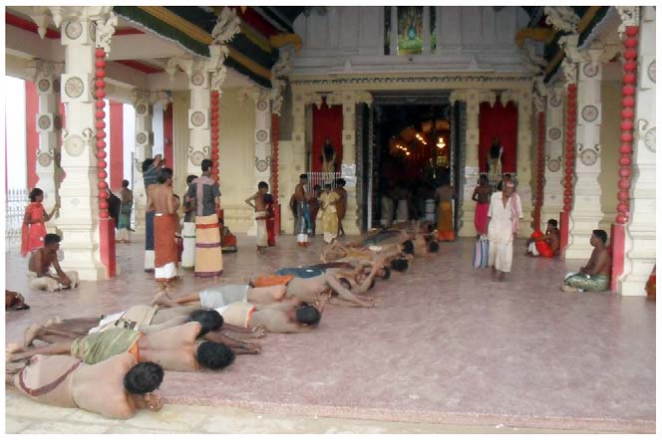
Devotees performing the ‘side-roll’ ritual.

During the festival period, which lasts for 25 days, large numbers of male devotees carry out a ritual known as the ‘side-roll’ in fulfillment of vows taken at the temple. In performing this ritual, men lie prostrate on the ground and roll sideways around the temple premises (a distance of approximately 600 m); their upper bodies are usually bare during this ritual (Figure 1). During the festival period, sand is brought from coastal areas by the Municipal Council and spread around the temple premises in order to reduce the devotees' discomfort. The sand is watered twice daily at about 3.00 am and 5.00 pm in order to keep the dust down.

This study was designed to find out the proportion of CLM among devotees carrying out the ‘side-roll’ ritual, their knowledge and practices regarding the condition, and the risk factors associated with infestation.

## Methods

### Ethics statement

By considering the religious sensitive issue, oral consent was obtained from the participants, instead of getting written consent to ensure the reliability of data, by explaining the nature and purpose of this research. Participants were given the freedom to withdraw from the study at any stage. The justification for getting oral consent was documented and submitted along with the methodology to the ethical review committee of the Faculty of Medicine, University of Jaffna, Sri Lanka. The committee has specifically approved the above study including the verbal consent procedure and issued the clearance certificate.

### Study design

A cross sectional analytical study was conducted among the devotees performing “side roll” during the festival at Nallur Temple, Jaffna, Sri Lanka.

Two hundred devotees were selected on the last day of the festival in August 2010, using systematic random sampling. Altogether around 1000 devotees would have been expected to do side role. Among the first five devotees, the 4^th^ devotee was selected randomly and thereafter, every 5^th^ devotee was systematically selected until 200 sample size was achieved. On the weekend, following the festival, selected devotees were asked to attend the Department of Parasitology in the Faculty of Medicine, Jaffna to provide the required information. Out of 200 selected devotees 194(97%) attended to the department.

### Data collection and analysis

Trained medical undergraduates applied a standardized questionnaire which covered the following four main areas i.e. a) demographic information; b) details of practice of side roll ritual; c) history of skin rash, its severity and treatment; d) awareness and knowledge of causative organism and precautionary measures practiced. After obtaining informed consent, questionnaires were administered in Tamil (the native langue of the devotees) by trained medical students. Study participants were also examined by them, and the itchy, erythematous, linear or serpiginous, dermatitis tract lesions were recorded on a pre-set form. Data was analyzed using statistical package SPSS version 17.

Bivariate analysis was done to identity the associated factors. Chi-squared test was used to find out the association and the odd ratio (OR) were calculated wherever possible. Then, binary logistic regression was performed. All possible factors indentified in bivariate analysis were included in the model. Those that contribute least to R^2^ are progressively removed until none of the remaining variables can be removed without leading to significant loss of R^2^ (Backward selection).In the final model, side role frequency and similar condition in the previous year were retained.

### Soil and faecal examination

In order to identify the source of infestation, 20 soil samples (two from each lot) were collected from the sand brought from the outside (coastal areas) before spreading it around the temple premises. The second set of 20 samples was collected from the temple premises 10 days after spreading the above sand. The above samples were examined at the Division of Parasitology, Department of Pathology, Faculty of Medicine, Jaffna. Baermann funnel technique was used for the recovery of nematode larvae from the above soil sample. The soil samples were added into a glass funnel which was fitted with a short piece of rubber tubing at the stem end. After 7 to 9 days, aliquots were drawn off by releasing the pinch of clamp on the rubber tubing and examined under the microscope. All five dog faecal samples, observed in the temple premises on a randomly selected day, were also examined for the presence of nematode eggs using wet smears in saline and iodine.

## Results

### Participant characteristics

Among all male devotees, who did the ritual side roll on the last day of the Nallur festival in 2010, 200 were included in this study. Six refused to participate on the ground of religious conviction. Background data of 194 devotees who participated in the study are shown in Table 1.

**Table 1:** Back ground details of participants (n = 194).

Variable	Categories	No of participants
**Age**	<19 yrs	23 (12%)
	20–40 yrs	146 (75%)
	>40 yrs	25 (13%)
**Education**	No formal education	2 (1%)
	Primary	5 (3)%
	Secondary	157 (81%)
	Tertiary	30 (15%)
**Occupation**	Student	45 (23%)
	Unemployed	8 (4%)
	Labourer	18 (9%)
	Farmer	15 (8%)
	Skill worker	38 (20%)
	Self employed	47 (24%)
	Teacher/Management assistant	20 (10%)
	Executive officer	3 (2%)
**Side roll frequency**	Every day	130 (67%)
	On selected days	64 (33%)
**Side roll time**	<5.00 a.m.	112 (58%)
	5.00 a.m. to 6.00 am	82 (42%)
**Side roll previous years**	Yes	173 (89%)
	No	21 (11%)
**Similar condition in previous years**	Yes	77 (40%)
	No	117 (60%)
**Awareness regarding the conditions**	Yes	157 (81%)
	No	37 (19%)
**Any precautionary measures taken**	yes	52 (27%)
	No	142 (73%)

### Performance of ‘side-roll’ ritual

Sixty seven percent of the respondents performed the ‘side-roll’ ritual every day (25 days), whereas 33% did it on selected, special days (10 out of 25 days). One hundred and twelve devotees (57.7%) stated that they carried out the side roll ritual before 5.00 am, whereas 82 (42.3%) stated that they did it between 5.00 and 6.00 am. A high proportion (89.2%) had done the side roll ritual in previous years too. Among these, 92 have performed the ritual consecutively for the last three or more years.

### Clinical features

**Figure 2:**
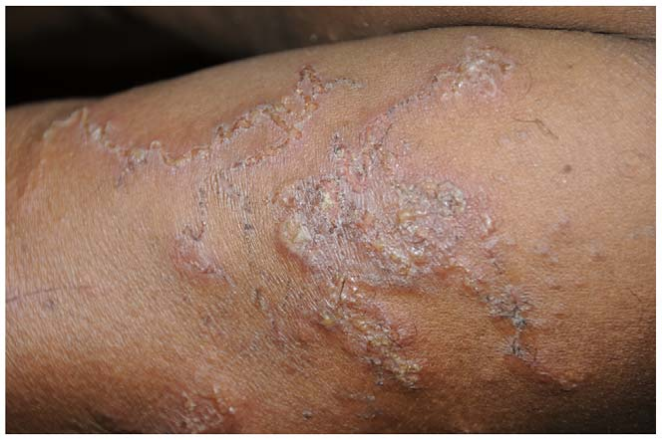
Typical cutaneous larva migrans lesion on a devotee's hand.

Among the 194 study participants, 58.2% (95% CI: 51.2%–65.0%) had lesions characteristic of CLM (Figure 2). Among those, 42.2% had the lesion only on the posterior aspects of the body including thorax, abdomen, arms and thighs. About three-fourth (79.6%) had more than one lesion. There was a moderate correlation (R^2^ = 0.446, P<0.001) observed between frequency of rolling and the number of lesions.

Among the participants 77 (36.7%) stated that they had a similar condition in previous years as well. Similar proportions of the study participants categorized the itchiness of the lesion as low (47.8%) or moderate (46.9%), while only a very small proportion (6.2%) categorized it as high. Thirty two (28.3%) study participants showed evidence of secondary bacterial infection of the CLM lesions. Study participants responded to occurrence of the condition in different ways. Interestingly, most of them (62%) treated themselves with soap and petrol. Another 23% stated that they sought treatment at private clinics where they were treated with a topical application cream (crushed tablet mixed with vaseline) and some oral drugs to minimize the itchiness.

### Awareness and precautionary measures of CLM

One hundred and fifty seven (80.9%) said that they were aware that devotees doing the side roll ritual at Nallur Temple were likely to develop skin conditions. Most had learnt this from their friends (92%). About half (51.5%) believed that the skin condition was caused by microbes in the soil, whereas 15.5% said that they did not know the cause of this condition.

Fifty two men (26.8%) stated that they had taken precautionary measures, the commonest (69%) being bathing immediately after doing the side roll ritual.

### Risk factors for infestation

Bivariate analysis of data showed significant association between occurrence of CLM lesions and frequency of performing the side roll ritual, the timing of ritual performance, awareness of the condition, had lesions in the previous years and use of precautionary measures (Table 2). However, logistic regression analysis identified frequency of ritual performance (OR-11.75; 95% CI 5.37–25.74) and similar conditions in previous years (OR-4.71; 95% CI: 2.14–10.39) were the independent risk factors.

**Table 2:** Risk factors associated with the infestation of Cutaneous Larva Migrans.

Risk factor	Category	CLM		Total	P Value	Odd Ratio with 95% CI
		Present No (%)	Absent No (%)			
**Side role frequency**	Every day	101(78%)	29(22%)	130	**P<0.001**	OR-15.10; 95% CI:7.12–32.01
	On selected days	12(19%)	52(81%)	64		
**Side role time**	Before 5am	73(65%)	39(35%)	112	**P = 0.022**	OR-1.96; 95% CI:1.10–3.52
	After 5am	40(49%)	42(51%)	82		
**Side role in previous year**	Yes	104(60%)	69(40%)	173	P = 0.13	OR-2.01; 95% CI:0.80–5.02
	No	9(43%)	12(57%)	21		
**Similar condition in the previous year**	Yes	64(83%)	13(17%)	77	**P<0.001**	OR-6.83; 95% CI:3.39–13.76
	No	49(42%)	68(58%)	117		
**Awareness regarding the condition**	Yes	99(63%)	58(37%)	157	**P = 0.005**	OR-2.80; 95%CI:1.34–5.87
	No	14(38%)	23(62%)	37		
**Precautionary measures taken**	Yes	33(63%)	19(37%)	52	P = 0.373	OR-1.35; 95% CI:0.70–2.60
	No	80(56%)	62(44%)	142		

### Environmental contamination

Twenty soil samples tested, before spreading it around the temple premises, were negative for nematode larvae. But, among the twenty samples examined, after the sand was spread, 2 were positive (10%) for the nematode larvae. Among the five samples of dog faeces collected from the temple premises, three were positive for hookworm eggs.

## Discussion

More than half of the population studied had CLM, which is almost twice when compared to the previous study conducted in the same location in 2002 [6]. This increase in the prevalence may be a result of flooding subsequent to the high rainfall experienced during the festival in 2010, which could have brought in dog faeces with hookworm eggs from neighboring areas. A similar observation was made recently in Brazil [7]. Further, the moisture could also have promoted the survival and dissemination of the larva [1]. Implementation of ban of killing stray dogs after 2004 may be an indirect cause.

As response rate is 97%, nonresponse bias is unlikely to be cause of this increase. Although the selection of last day might be a potential source of bias, which could be considered as minimal, since almost all the devotees select the last day as it is the very special day to perform side roll. Participants from the final day were selected by using systematic random sampling. Hence, it fairly represents the all devotees who did the side rolling on that day.

The lesions observed on the exposed parts of the body which has most contact with the ground during performance of the ritual indicates, that the reflecting parts of the skin has the higher risk of getting CLM. In a study during one outbreak in Canadian tourists, it was reported that less frequent use of protective footwear while walking on the beach was significantly associated with a higher risk of developing the disease [8].

The frequency of side role, side role time, performed side role in previous years, had similar condition in previous year, awareness regarding this condition and any precautionary measure were identified as the risk factors among the devotees performed the side roll ritual activities.

Combinations of the above factors resulted in the high prevalence of infestation among these devotees. The frequency of performing ritual activities and CLM infestation in the previous years were the two factors found to be of significance in the multivariate analysis. Increased frequency in performance of the ritual evidently increases the period of contact between bare skin and soil, and therefore increases risk of infestation. The environmental conditions were also highly suitable for the survival of hookworm larvae in the sand which is contaminated with dog faeces. Hookworm larvae are known to survive better in sandy soil [8]–[10], which is brought from coastal areas by the Municipal Council. High moisture content of the soil further enhances the larval survival [9], occurred either by rain or by sprinkling the water twice a day on the soil.

Those who performed the ritual earlier in the morning had a higher rate of infestation than those who did so later on. A study carried out among travelers in island of Barbados, showed that walking on the beach at night showed trends towards higher risk of acquiring the CLM [10]. Those who had awareness of the occurrence of CLM among devotees had a significantly higher incidence of infestation than those who did not aware about it. The most likely explanation for this combination of results is that those who perform the ritual most frequently are also those who do it earlier in the morning and those who have greater awareness of the accompanying problems. The statistical analysis also indicated that the precautionary measures practiced by devotees do not in fact reduce the risk of incidence.

Regarding the treatment for this condition it was observed that majority of them treating either by themselves or seeking the private clinics. Although the drug albendazole is recommended for the treatment of CLM, many participants stated that they have used mebendazole with vaseline for the local application. Since the participants were familiar with the drug mebendazole as the routine deworming drug, either they confused the drug albendazole given by the practitioners as Mebendazole or really it was the case. It indicates the importance of awareness among the participants regarding treatment and precautionary measures against CLM.

There are a few preventive measures that could reduce the incidence of CLM among devotees in future festivals at the Nallur Temple. The most logical of these would be to secure the temple premises in order to prevent dogs from accessing the grounds and defaecating on the sand. However, this may be difficult to implement, since the temple premises are not walled off completely at present and stray dogs are numerous. Reduction in the frequency with which the sand is watered could reduce soil moisture and thereby, the larval survival. However, this needs to be balanced against the need to keep down the dust. Another measure that could prevent infestation is to minimize contact between bare skin and sand by the use of some kind of upper body apparel. However, this may be in conflict with the religious tradition and therefore be unacceptable. The role of religious convictions in this situation is exemplified by the six devotees who refused to participate in this study on the ground they believed that getting this condition is evidence of the grace of the God and that the number of lesions is proportionate to the grace.

In conclusion, performance of the side-roll ritual at the Nallur festival exposes devotees to a high risk of acquiring CLM due to contamination of the sand in the temple premises with dog faeces.

Frequency of ritual performance and similar conditions in previous years were identified as the most significant independent risk factors. Among the above two factors, it is very difficult to control the frequency of ritual performance due to the religo-cultural reasons. At the same time, the association between past history and current CLM need to be studied further by follow up study. Therefore, deworming the stray dogs around the temple premises will be an effective and feasible precautionary measure to control similar epidemics in future. Moreover, creating the awareness among the public regarding CLM, is also important.
